# Functional transcriptomic annotation and protein–protein interaction network analysis identify NEK2, BIRC5, and TOP2A as potential targets in obese patients with luminal A breast cancer

**DOI:** 10.1007/s10549-017-4652-3

**Published:** 2018-01-12

**Authors:** Miriam Nuncia-Cantarero, Sandra Martinez-Canales, Fernando Andrés-Pretel, Gabriel Santpere, Alberto Ocaña, Eva Maria Galan-Moya

**Affiliations:** 1 Translational Oncology Laboratory, Centro Regional de Investigaciones Biomédicas (CRIB), Universidad de Castilla La Mancha (UCLM), C/Almansa 14, 02008 Albacete, Spain; 2 Translational Research Unit, University Hospital, Albacete, Spain; 3 Department of Neuroscience, Yale School of Medicine, New Haven, CT USA

**Keywords:** Breast cancer, Transcriptomic analysis, Protein–protein interaction, Clinical outcome, Novel druggable targets, Targeted therapy

## Abstract

**Purpose:**

Although obesity is a risk factor for breast cancer, little effort has been made in the identification of druggable molecular alterations in obese–breast cancer patients. Tumors are controlled by their surrounding microenvironment, in which the adipose tissue is a main component. In this work, we intended to describe molecular alterations at a transcriptomic and protein–protein interaction (PPI) level between obese and non-obese patients.

**Methods and results:**

Gene expression data of 269 primary breast tumors were compared between normal-weight (BMI < 25, *n* = 130) and obese (IMC > 30, *n* = 139) patients. No significant differences were found for the global breast cancer population. However, within the luminal A subtype, upregulation of 81 genes was observed in the obese group (FC ≥ 1.4). Next, we explored the association of these genes with patient outcome, observing that 39 were linked with detrimental outcome. Their PPI map formed highly compact cluster and functional annotation analyses showed that cell cycle, cell proliferation, cell differentiation, and cellular response to extracellular stimuli were the more altered functions. Combined analyses of genes within the described functions are correlated with poor outcome. PPI network analyses for each function were to search for druggable opportunities. We identified 16 potentially druggable candidates. Among them, NEK2, BIRC5, and TOP2A were also found to be amplified in breast cancer, suggesting that they could act as strategic players in the obese-deregulated transcriptome.

**Conclusion:**

In summary, our in silico analysis describes molecular alterations of luminal A tumors and proposes a druggable PPI network in obese patients with potential for translation to the clinical practice.

**Electronic supplementary material:**

The online version of this article (10.1007/s10549-017-4652-3) contains supplementary material, which is available to authorized users.

## Introduction

Breast cancer is the leading cause of cancer-related death among women worldwide [1]. To this regard, several factors are involved in the initiation and promotion of breast tumors including molecular alterations at the genomic level such as mutations or copy number alterations [2, 3]. Indeed, using functional studies, some of these genomic modifications have been clearly associated with a malignant phenotype, contributing to the oncogenesis of epithelial cells [4, 5]. In addition to these molecular alterations, cancer cells rely on the surrounding microenvironment, where non-transformed cells and stromal components facilitate tumor growth by the secretion of autocrine signals like growth factors [6]. Stimulation of cancer cells by paracrine-secreted factors from interstitial cells including fibroblasts, neutrophils, or endothelial cells can stimulate functions such as proliferation, survival, or migration, which are necessary to the tumor formation and dissemination [7, 8]. However, components of the tumor stroma depend on different conditions and can differ among individuals. Of note, adipose tissue is one of the main components of the breast cancer microenvironment, and therefore, accumulation of fat tissue in the stroma can modify settings of tumor cells and influence their survival [8]. As an example, increased presence of insulin or insulin-like growth factors can affect tumor growth but also response to treatment [9]. In this context, breast tumors that express estrogen receptors are more dependent on stimulating factors [10].

Besides being a risk factor for cancer, obesity has also been associated with detrimental patient outcome, especially in postmenopausal patients [5, 4]. A number of epidemiological studies have demonstrated that how obesity is directly related to cancer mortality. In this sense, an increased body mass index (BMI) has been strongly linked with poor survival in postmenopausic patients carrying estrogen receptor positive tumors [11]. One of the mechanisms proposed to explain how obesity increases breast cancer risk is that adipocyte-secreted hormones could be promoting tumor progression through an increase of cellular proliferation [12]. However, little effort has been put into clarifying how the excess of adipose tissue in the tumor niche influences the molecular characteristics of the residing malignant cells.

In the present article, we aimed to evaluate biological functions that differentiate breast cancer tumors from obese and non-obese patients. To do so, we performed transcriptomic followed by protein–protein interaction network analyses to recognize relevant biological functions with druggable implications.

## Materials and methods

### Transcriptomic and gene expression analyses using bioconductor

We used a public data set (GEO Data Set accession number: GSE 78958) to compare mRNA levels from 405 breast cancer tumors. Affymetrix CEL files were downloaded and analyzed with R 3.3.2 (Bioconductor package). Data from patients not matching our BMI criteria (BMI < 25 or ≥ 30) were excluded from the analysis, what reduced tumor samples to 269. Before proceeding with the comparative analysis, we performed a statistical quality control (QC), including relative log expression (RLE) and normalize unscaled standard error (NUSE) graphs. QC validated all samples for the following comparative analysis. Normalization was performed using the robust multi-array (RMA) system, included in the affymetrix package, and screening with the genefilter package. Data comparison was done using the limma package, comparing array data from each patient group (normal weight: BMI < 25; obese: BMI ≥ 30). Once the matrix for the experimental design was established, we used the function lmFit to perform a linear adjustment and create the contrast matrix in agreement with the compared groups, necessary to accomplish the Bayesian adjustment to determine the final fold change.

### Analysis of patient characteristics

Comparison of proportions between normal-weight and obese groups we performed for each variable including grade, TNM stage, and patient ethnicity. This comparison was fulfilled using either Pearson’s Chi-square or Fisher’s exact test; *p < 0.05, **p < 0.001.

### Construction and analysis of PPI networks and functional annotation

We used the online tool STRING (http://www.string-db.org) to construct interactome maps of deregulated genes (STRING v10 data accessed: 14/02/17 and 10/07/17). Thus, we constructed a PPI map for the underexpressed genes and another for the overexpressed genes. The indicated network properties include:

*Nodes*: number of proteins in the network; *Edges:* number of interactions; *Node degree:* average number of interactions; *Clustering coefficient:* indicates the tendency of the network to form clusters. The closer the local clustering coefficient is to 1, the more likely it is for the network to form clusters; *PPI enrichment p value:* indicates the statistical significance. Proteins are considered hubs when they have more interactions than the average (nº interactions > node degree).

Functional screening for overexpressed genes was performed using Ensembl database (http://www.ensembl.org).

### Evaluation of clinical outcome

The free-access Kaplan–Meier (KM) Plotter Online Tool (http://kmplot.com/analysis/) was used to investigate the relationship between gene expression levels and patient’s clinical outcome in luminal A breast cancer. Only overexpressed genes significantly associated with detrimental outcome (Hazard Ratio > 1 and *p* value ≤ 0.05) were used for subsequent analysis (*n* = 39). This tool was also used to determine relapse free survival (RFS) and overall survival (OS) in combined analyses of all genes included within cell cycle, cell differentiation, cell proliferation, and cellular response to extracellular stimuli functions. All the analyses were performed independently by two authors (MNC and SMC) and reviewed by a third author (EMGM). No discrepancies were observed.

### Identification of drug candidates

We initially used data from The Drug Gene Interaction Database (DGIdb) http://dgidb.genome.wustl.edu/ to identify potentially druggable genes and their associated drugs among PPI hub proteins. Next, we used information from Genecards (www.genecards.org), which contains information from several databases, to manually select further druggable target among the PPI hub proteins of each function. Then, as described above, we used the STRING tool to build the druggable obese interactome.

### Molecular alteration identification

We used data contained at cBioportal (www.cbioportal.org), Breast Invasive Carcinoma TCGA, *n* = 816 [13], to identify potential copy number alterations (amplification or deletion) and the presence of mutations in the druggable genes.

## Results

### Differential gene expression between normal-weight and obese breast cancer patients with luminal A tumors

**Fig. 1:**
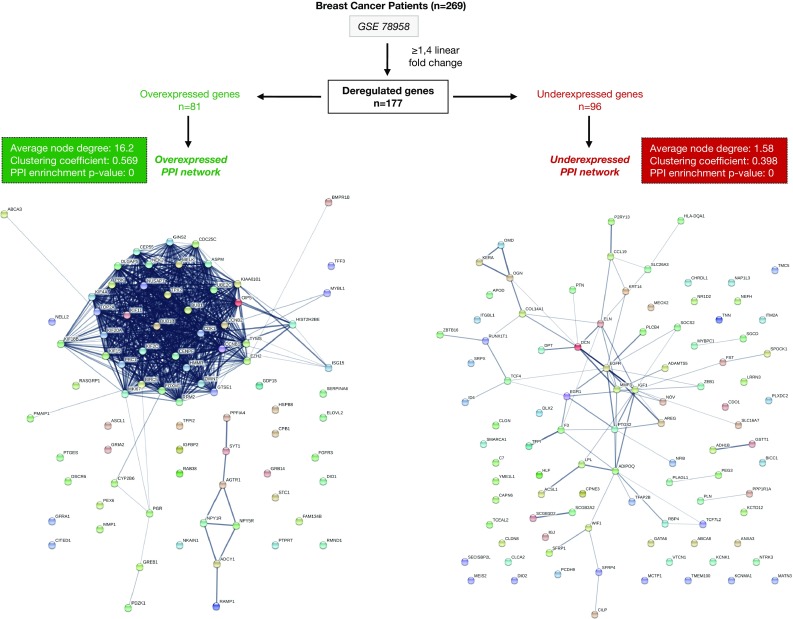
Gene expression comparison between normal-weight and obese luminal A breast cancer patients. We identify 177 deregulated genes (fold change ≥ 1.4) in luminal A obese patients. Then, using the online tool STRING, PPI networks for the underexpressed (*n* = 96) and the overexpressed (*n* = 81) genes were constructed

We performed gene expression analyses in a cohort of 269 breast cancer patients based on their body mass index (BMI). The initial comparison between normal-weight (*n* = 130) and obese (*n* = 139) patients including all breast cancer tumors did not show statistical differences between both groups. Therefore, we decided to perform the analysis in each intrinsic tumor specific subtype (basal-like, HER2, luminal A, and luminal B). While no significant differences were found for the Basal-like, HER2, and luminal B subtypes, we identified 177 deregulated genes in the luminal A subtype (Table 1). Using a fold change of 1.4, we selected 96 and 81 genes that were underexpressed and overexpressed, respectively, in the obese group (Fig.1 and Table S1). Of note, the analysis of patient characteristics showed no significant differences between groups in relation with clinical stage or tumor grade. However, African American women displayed a significant higher proportion of obese women (Table 2).

**Table 1:** Gene expression comparison between normal-weight (B.M.I < 25) and obese (B.M.I ≥ 30) breast cancer patients for each molecular subtype

Breast cancer patients (GSE 78958; *n* = 269)			
Intrinsic subtypes groups	Normal weight (B.M.I < 25)	Obese (B.M.I ≥ 30)	Deregulated genes
Basal-like (*n* = 64)	*n* = 30	*n* = 34	NS
HER2-enriched (*n* = 27)	*n* = 15	*n* = 12	NS
Luminal A (*n* = 145)	*n* = 68	*n* = 77	177
Luminal B (*n* = 33)	*n* = 17	*n* = 16	NS

**Table 2:** Proportion comparison of patients’ clinical characteristics based on patients BMI

	% (*n*)		*p* value
	Normal weight < 25 (*n* = 68)	Obese ≥ 30 (*n* = 77)	
**Grade**			
Well (Grade 1)	50.9% (27)	49.1% (26)	0.182
Moderate (Grade 2)	48.7% (37)	51.3% (39)	
Poor (Grade 3)	25.0% (4)	75% (12)	
**TNM Stage**			
Stage I	53.0% (35)	47.0% (31)	0.886
Stage II	42.6% (26)	57.4% (35)	
Stage III	37.5% (6)	62.5% (10)	
Stage IV	50.0% (1)	50.0% (1)	
**Ethnicity**			
African American	30.0% (9)	70.0% (21)	0.008
European American	48.6% (53)	51.4% (56)	
Other	100.0% (6)	0.0% (0)	

Next, we constructed protein–protein interaction (PPI) maps of both identified groups and analyzed the potential functional modules within the networks. Notably, we found a higher number of interactions among proteins in the overexpressed PPI network (node degree: 16.2; clustering coefficient: 0.569) when compared with the underexpressed (node degree: 1.58; clustering coefficient: 0.398). Indeed, while proteins in the underexpressed map did not exhibit any cohesion, the overexpressed PPI network contained a marked cluster unit (Fig.1). Functional annotation analyses of the overexpressed genes identified 32 biological functions (Fig. S1 and Table S2).

### Identification of upregulated genes associated with worse outcome

**Fig. 2:**
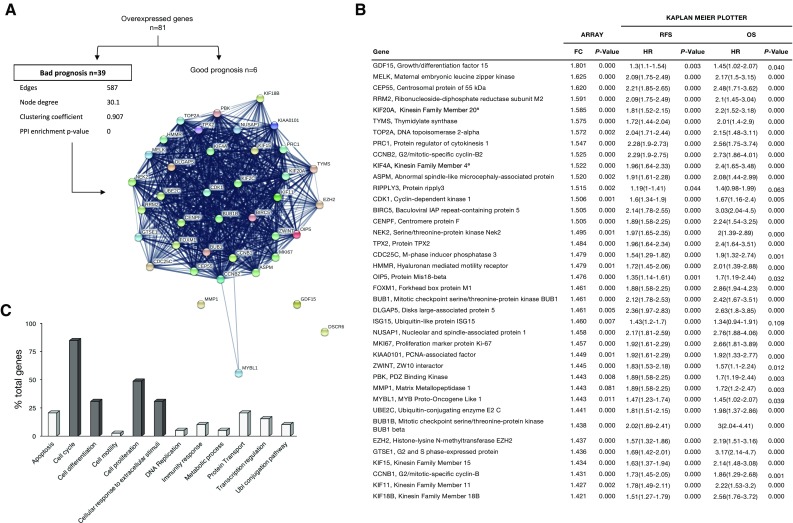
PPI map and functional annotation of bad prognosis-associated upregulated genes in luminal A breast cancer obese patients. **a** Using the K–M plotter tool, we identified overexpressed genes associated with bad prognosis and used them to construct a network of detrimental outcome in luminal A obese patients. **b** List of overexpressed genes associated with bad prognosis. Probe and transcript ID together with the symbol are indicated for each gene. Table includes the information for specific fold change difference, Hazard ratio (HR) for relapse free survival (RFS), and overall survival (OS). **c** Functional enrichment analyses of bad-prognosis-associated genes identifies cell cycle, cell differentiation, cell proliferation, and cellular response to extracellular stimuli as the most altered functions in luminal A obese patients

Next, we intended to identify the role of the 81 overexpressed genes in relation with patient outcome. We used data contained in the KM plotter online tool [14] that enclose information for more than 5000 breast cancer patients. We identified that 39 genes were significantly associated with detrimental patient outcome, including relapse free survival (RFS), overall survival (OS), or both (Fig. 2a, b), in the subgroup of luminal A breast tumors. To investigate how these genes are interacting among them, we constructed their PPI network. Notably, the PPI map of the proteins codified by the bad prognosis-associated genes formed a highly interconnected cluster (node degree: 30.1; clustering coefficient: 0.901) (Fig. 2a), suggesting that they could act as components of a protein complex with functional links.

### Functional annotation analyses of worse outcome

We performed functional annotation analyses of the genes which predicted unfavorable outcome to identify biological functions that led to a high clustering coefficient [15]. In line with our previous result, many of these genes participated in multiple functions and were, therefore, included in more than one functional group (Table S3). We identified 12 biological functions (Fig. 2c), being cell cycle (33 genes), cell proliferation (19 genes), differentiation (12 genes), and cellular response to extracellular (EC) stimuli (12 genes) the most represented.

**Fig. 3:**
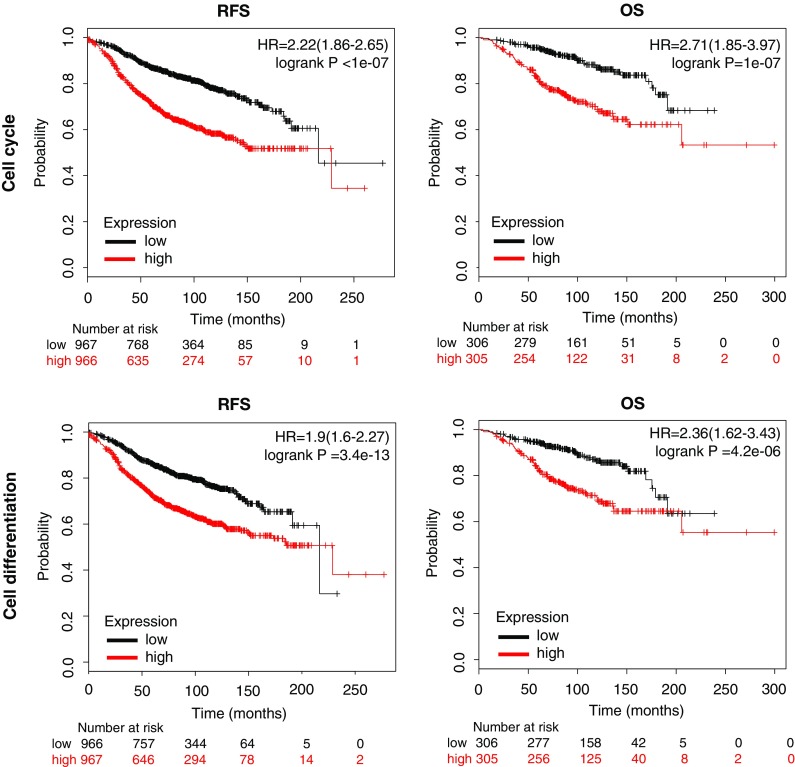
Association with relapse free survival (RFS) and overall survival (OS) of gene sets included in the cell cycle and cell differentiation functions in luminal A breast cancer

**Fig. 4:**
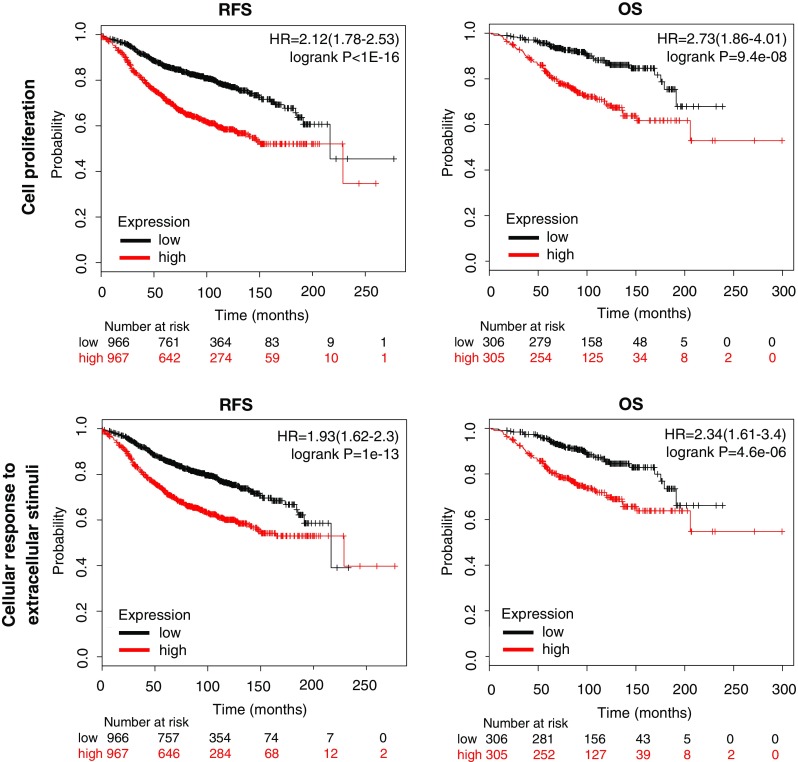
Association with relapse free survival (RFS) and overall survival (OS) of gene sets included in the cell proliferation and cellular response to extracellular stimuli functions in luminal A breast cancer

The combined analysis of genes contained in each of the functions was associated with detrimental RFS and OS in luminal A (Figs. 3, 4). Cell cycle showed the best correlation with RFS [HR = 2.22 (1.86–2.65), log rank *p* < 1e-07] and a significant association with detrimental OS [HR = 2.71 (1.85–3.97), log rank *p* = 1e-07] (Fig. 3). Cell differentiation gene cluster predicts poor RFS [HR = 1.9 (1.6–2.27), log rank *p* = 3.4e-13] and OS [HR = 2.36 (1.62–3.43), log rank *p* = 4.2e-06] (Fig. 3). Cell proliferation was also associated with lower RFS [HR = 2.12 (1.78–2.53), log rank *p* < 1e-16] and demonstrated the strongest association with poor OS [HR = 2.73 (1.86–4.01), log rank *p* = 9.4e-08] (Fig. 4). Finally, cellular response to EC stimuli gene showed poor RFS [HR = 1.93 (1.62–2.3), log rank *p* = 1e-13] and OS [HR = 2.34 (1.61–3.4), log rank *p* = 4.6e-06] (Fig. 4). We also explored the potential of these functional groups to predict patient outcome in the other molecular subtypes. Of note, combined analysis of these functional genes groups in Luminal B, HER2 and basal-like subtypes poorly or no significantly correlated with prognosis (Table S4).

### NEK2, BIRC5, and TOP2A are potential therapeutic targets in luminal A obese patients

Protein interactions can be used to prioritize gene candidates in in silico studies and to identify potential druggable targets [16]. PPI networks for these four functions confirmed their functional clustering unity (Fig. S2) and uncovered 18, 10, 6, and 5 hub proteins for cell cycle, cell proliferation, cellular response to extracellular stimuli, and cell differentiation, respectively (Table S5). Of note, BUB1 and CDK1 were the components showing more interactions (edges).

**Fig. 5:**
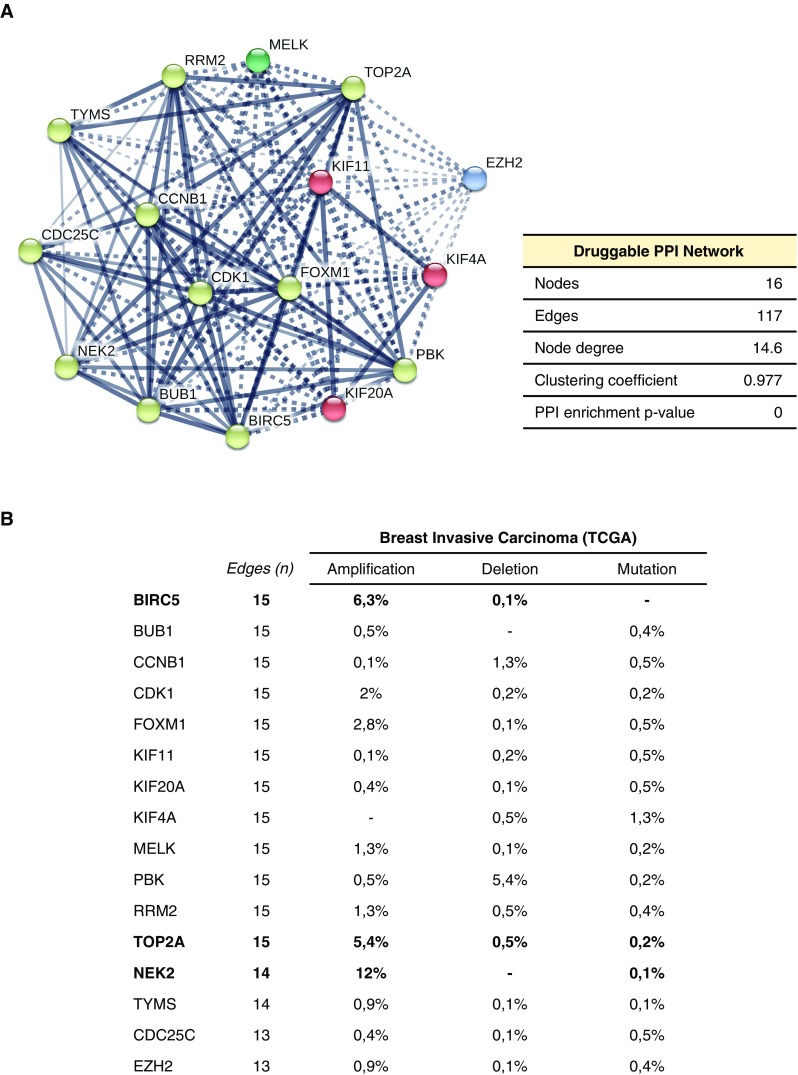
Druggable PPI network in luminal A obese patients. **a** We used the online tool STRING to construct the PPI network of the druggable targets. **b** Percentage of copy number alterations (amplifications and deletions), and presence of mutation for each druggable gene, obtained from cBioportal data

Once we had identified core proteins for each functional cluster, we searched for druggable targets within the networks. Based on their interaction with existing drugs and/or the existence of chemical inhibitors, we identified 16 potentially druggable candidates (Supplementary Table 6). We used the identified targetable proteins to construct a druggable PPI map (Fig. 5a). The resulting druggable network has a clustering coefficient tightly close to 1 (0.977), with 12 out of the 16 nodes showing interactions with all the network components (node degree ≥ 15), what support the idea of a biological functional unit. Thus, the use of compounds against any node within the system might potentially affect the whole network, producing a wider response.

To complete our study, we searched for information about copy number alterations or mutations of the 16 identified druggable genes in the cancer genomics database (cBioportal, [13, 17]. Deletions and mutations were present at a very low frequency. However, NIMA (Never in Mitosis Gene A)-Related Kinase 2 (*NEK2*), Apoptosis Inhibitor Survivin (*BIRC5*) and Topoisomerase (DNA) II Alpha (*TOP2A*) were found to be amplified in breast cancer (12, 6.3, and 5.4%, respectively) (Fig. 5b), suggesting a high potential role for them as therapeutic targets in luminal A patients.

## Discussion

In the present article, we describe biological functions and PPI networks associated with obesity in luminal A tumors, which we found generally associated with worse outcome in luminal A patients. Moreover, we uncover a druggable PPI network on luminal A obese patients which could be of utility to design potential therapeutic strategies.

Although we initially investigated all four subtypes of breast cancer, we only found significant differences between normal-weight and obese patients for the luminal A subtype.

Although this might be due to the fact that luminal A group was the most abundant, the relevant association with outcome and the strong PPI network suggests a relevant biological role. Moreover, the combined analysis of genes within each function was associated with poor outcome in luminal A patients. Of note, the subgroup analysis did not correlate with patient outcome in the other three molecular subtypes (luminal B, HER2, and basal-like). This supports the idea that deregulation of these functions has a specific role in luminal A breast cancers. Our results are in line with those obtained in another study using the same transcriptomic database (GSE 78958) [18], although in this study, PPI analysis and druggable target opportunities were not explored. In addition, our study also identified a signature that is specific for luminal A tumors and explored the relation of this signature with patient outcome.

Although the level of deregulation between obese and non-obese patients was not highly elevated, we found significant differences using a fold changed of 1.4. We are aware that the fold change used is smaller than the one used in other studies [19, 20]. However, this finding could be explained by the fact that obese patients included in our study lack the exact BMI information. It could be expected that the exclusion of the obese-I subtype might have been of utility to increase the difference among our study groups. In any case, even the number of deregulated genes and the level was not high, the upregulation of these transcripts was associated with an important detrimental outcome.

The most frequent functions identified in the overexpressed genes and linked with worse outcome included: cell cycle, cell differentiation, cell proliferation, and cellular response to EC stimuli. Control of cell cycle was the most frequent pathway with genes involved in the formation of the mitotic spindle and centrosome or microtubules formation like *BUB1, NUSAP, CENPF, CEP55,* or the *KIF* family [21]. In addition, other genes were associated with the regulation and control of the cell cycle such as *CDK, GTSE1, CDC25C,* or *CCNB* [22]. Finally, *FOXM1*, a transcription factor linked with the presence of a Luminal phenotype, was found to be upregulated [23]. Of note, our study also uncovered some interesting downregulated genes in the obese group. Notably, while *EGFR* is overexpressed in around 50% TNBC and inflammatory breast cancers [24], we found that this gene, as well as its ligand *AREG*, was downregulated in the luminal A obese group. Wnt signaling has been implicated in carcinogenesis as well as in obesity promotion [25, 26]. In this line, luminal A obese group also showed a lower expression of Wnt pathway inhibitors, such as *WIF1, BICC1,* and the secreted proteins *SFRP1* and *SFRP4*, together with a downregulation of the negative regulators of MYC, *TFAP2B,* or *TCF7L* [27, 28].

Protein–protein interaction (PPI) networks offer information of how different proteins cooperate with others to trigger biological processes within the cell [15, 29]. In this context, we have constructed PPI networks for the deregulated genes in luminal A obese patients. While proteins coded by deregulated genes poorly interact, we have found that exist a solid-clustering unit within the overexpressed PPI network. Remarkably, this dense cluster was comprised by proteins specifically coded by overexpressed genes that were associated with detrimental patient outcome. Thus, interference of one of its components might have an impact on several nodes, which could in turn lead to the destabilization of the network. This could open the window to new therapeutic strategies targeting this overexpressed PPI network in luminal A obese patients.

Next, we decided to search for potential drug targets within the PPI networks, linked with poor prognosis. Using Drug Interaction Database [30], we first identified eight druggable genes: BUB1, TOP2A, BIRC5, KIF11, NEK2, RRM2, TYMS, and PBK. Then, expanding the search to other drug databases, we added eight more druggable candidates: CCNB1, CDK1, FOXM1, KIF4A, KIF20A, MELK, NEK2, and CDC25C. The PPI network built with them exhibited a high degree of interactions and, as indicated by its high clustering coefficient, might act as a cohesive functional unit.

Mitotic-related targets in this druggable network are the aim of new chemical entities with potential for preclinical or clinical translation development [31]. For instance, a well-described target is TOP2A, where doxorubicin-like chemotherapies inhibit their effect [32, 33]. Similarly, strategies to target BUB1 are under preclinical evaluation as this kinase has been described as associated with detrimental prognosis in breast and ovarian cancer [20, 34]. Compounds against KIF11 are under development [35] and some in clinical development [36].

BIRC5 codifies for a protein which is vital for the growth and survival of cancer cells. Survivin is found to be essential for several functions linked with oncogenic transformation [37]. It is known that normal tissues do not express survivin, and high expression in tumors is an indicative of poor prognosis and intrinsic resistance to radio- and chemotherapy [38]. As obese patients express high levels of BIRC5, evaluation of BIRC5 inhibitors, alone or in combination, could be a potential option.

Finally, NEK2 codifies for a serine–threonine kinase with a key role in mitosis that has been found to be aberrantly overexpressed in several cancer types, among them breast cancer [39, 40]. NEK2 expression levels are associated with tumor progression and detrimental outcome, as well as with drug resistance [41]. Besides, preclinical studies have shown that high NEK2 levels can induce tumorigenesis by mediating chromosome instability and aneuploidy, while its downregulation can lead to cancer cells death [42], suggesting a role for NEK2 as a potential target to treat cancer. Its elevated levels in obese patients points at NEK2 as a good candidate for targeted therapy in these patients. However, although several inhibitors have been developed for NEK2, some of them being highly specific and showing an irreversible inhibition, they have not been taken to clinical evaluation yet [43].

Notably, NEK2 as well as BIRC5 and TOP2A were amplified in more than 12, 6, and 5% of breast cancers, respectively, reinforcing their potential role as key therapeutic targets.

In conclusion, in the present work, we describe functional pathways and protein–protein interacting networks associated with clinical outcome in luminal A tumors from obese patients. Moreover, we identify a druggable interacting map with potential for target inhibition. Although we acknowledge that this is an in silico analyses, and data should be confirmed in samples from patients, our results open new venues for further characterization and have potential for translation into the clinical setting.

## Electronic supplementary material

Below is the link to the electronic supplementary material.

Supplementary material 1 (PDF 8989 kb)

Supplementary material 2 (DOCX 71 kb)

Supplementary material 3 (PDF 253 kb)

Supplementary material 4 (PDF 5171 kb)

Supplementary material 5 (PDF 52 kb)

Supplementary material 6 (PDF 130 kb)

Supplementary material 7 (PDF 78 kb)

Supplementary material 8 (PDF 39 kb)

Supplementary material 9 (PDF 22 kb)

Supplementary material 10 (PDF 101 kb)
