# Supplementary material for: Functional transcriptomic annotation and protein–protein interaction network analysis identify NEK2, BIRC5, and TOP2A as potential targets in obese patients with luminal A breast cancer
Source: Breast Cancer Res Treat. 2018 Jan 12;168(3):613–23. doi: 10.1007/s10549-017-4652-3 (PMC5842257; doi:10.1007/s10549-017-4652-3)
Supplement: Supplementary file 1 — Supplementary material 1 (PDF 8989 kb) [file 10549_2017_4652_MOESM1_ESM.pdf]

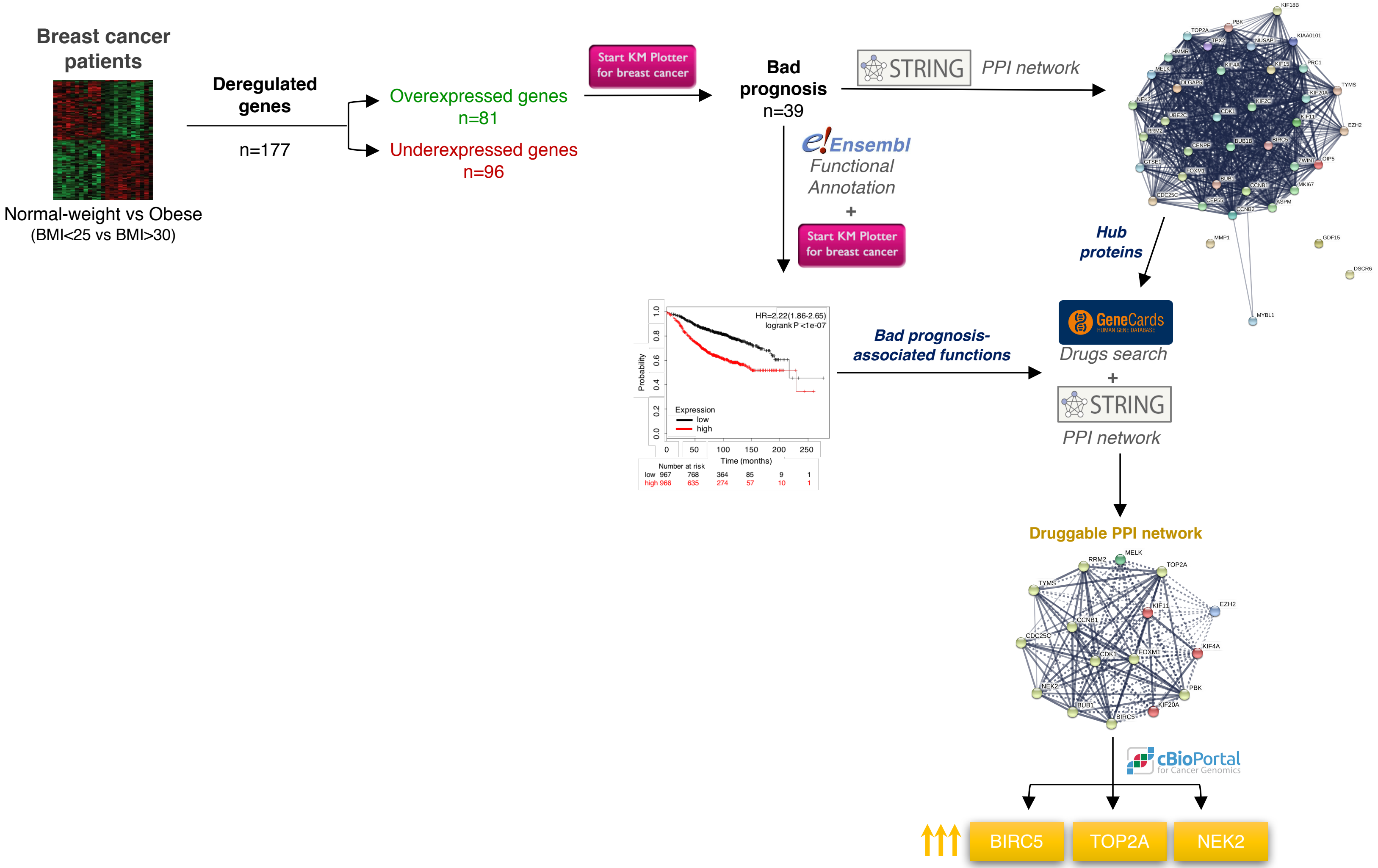
Breast cancer patients
Normal-weight vs Obese
(BMI<25 vs BMI>30)
Deregulated genes
n=177
Overexpressed genes
n=81
Underexpressed genes
n=96
Start KM Plotter for breast cancer
Bad prognosis
n=39
STRING
PPI network
Ensembl
Functional Annotation
+
Start KM Plotter for breast cancer
HR=2.22(1.86-2.65)
logrank P <1e-07
Probability
Expression
low
high
Time (months)
Number at risk
low 967 768 364 85 9 1
high 966 635 274 57 10 1
Bad prognosis-associated functions
Hub proteins
GeneCards
HUMAN GENE DATABASE
Drugs search
+
STRING
PPI network
Druggable PPI network
RRM2
MELK
TOP2A
TYMS
EZH2
KIF11
CCNB1
CDC25C
KIF4A
FOXM1
CDK1
NEK2
PBK
BUB1
KIF20A
BIRC5
cBioPortal
for Cancer Genomics
BIRC5
TOP2A
NEK2
