# Supplementary material for: Functional transcriptomic annotation and protein–protein interaction network analysis identify NEK2, BIRC5, and TOP2A as potential targets in obese patients with luminal A breast cancer
Source: Breast Cancer Res Treat. 2018 Jan 12;168(3):613–23. doi: 10.1007/s10549-017-4652-3 (PMC5842257; doi:10.1007/s10549-017-4652-3)
Supplement: Supplementary file 2 — Supplementary material 2 (DOCX 71 kb) [file 10549_2017_4652_MOESM2_ESM.docx]

**Supplementary Table 1.** List of overexpressed and underexpressed genes in luminal A obese patients.

**Supplementary Table 2.** Functional annotation of overexpressed genes in luminal A obese patients.

**Supplementary Table 3.** Functional annotation of bad prognosis-associated overexpressed genes in luminal A obese patients.

**Supplementary Table 4.** Association with relapse free survival (RFS) and overall survival (OS) of gene sets of the main four identified functions in Luminal A and B, HER2 and TNBC.

**Supplementary Table 5.** List of hubs proteins for the four predominant functions.

**Supplementary Table 6.** List of potential druggable targets and associated drugs

**Supplementary Figure 1.** Underexpressed and overexpressed PPI networks in luminal A obese patients.

**Supplementary Figure 2.** Protein-protein interaction maps of the four outstanding functions.
