# Supplementary figures and images for: Functional transcriptomic annotation and protein–protein interaction network analysis identify NEK2, BIRC5, and TOP2A as potential targets in obese patients with luminal A breast cancer
Source: Breast Cancer Res Treat. 2018 Jan 12;168(3):613–23. doi: 10.1007/s10549-017-4652-3 (PMC5842257; doi:10.1007/s10549-017-4652-3)

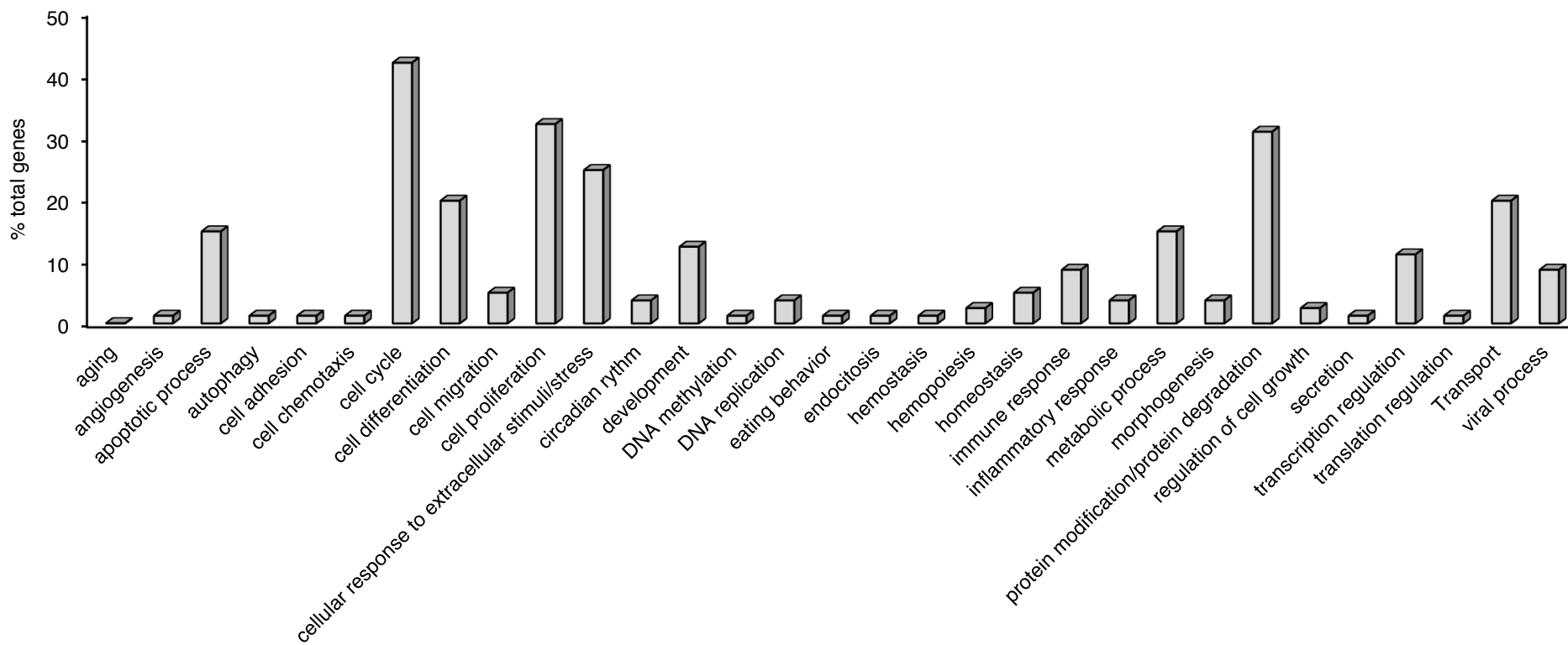

Supplement: Supplementary file 3 — Supplementary material 3 (PDF 253 kb) [file 10549_2017_4652_MOESM3_ESM.pdf]
