# Supplementary material for: Functional transcriptomic annotation and protein–protein interaction network analysis identify NEK2, BIRC5, and TOP2A as potential targets in obese patients with luminal A breast cancer
Source: Breast Cancer Res Treat. 2018 Jan 12;168(3):613–23. doi: 10.1007/s10549-017-4652-3 (PMC5842257; doi:10.1007/s10549-017-4652-3)
Supplement: Supplementary file 4 — Supplementary material 4 (PDF 5171 kb) [file 10549_2017_4652_MOESM4_ESM.pdf]

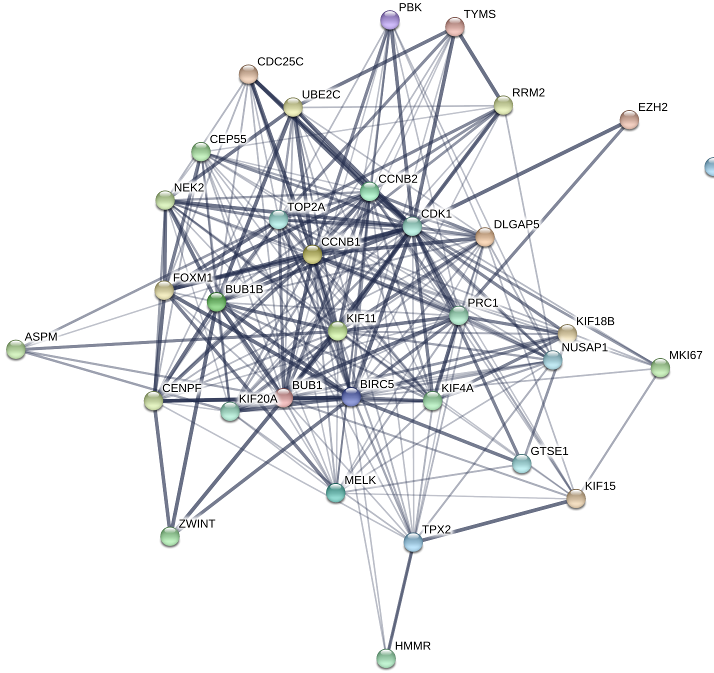

Cell cycle	
Nodes	33
Edges	238
Node degree	14.4
Clustering coefficient	0.755
PPI enrichment p-value	0

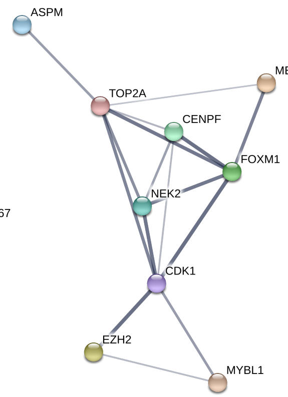

Cell differentiation	
Nodes	12
Edges	16
Node degree	2.67
Clustering coefficient	0.886
PPI enrichment p-value	1.39e-06

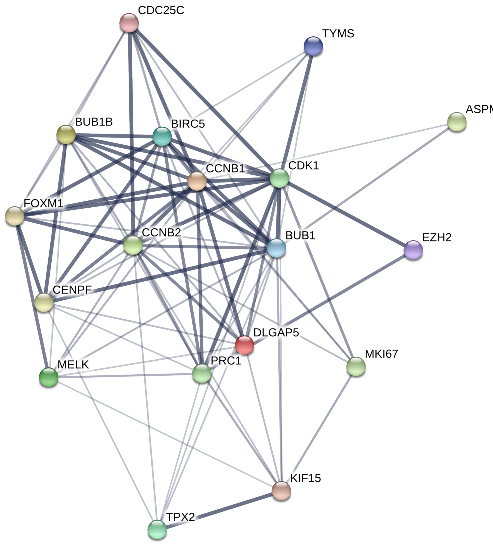

Cell proliferation	
Nodes	19
Edges	83
Node degree	8.74
Clustering coefficient	0.809
PPI enrichment p-value	0

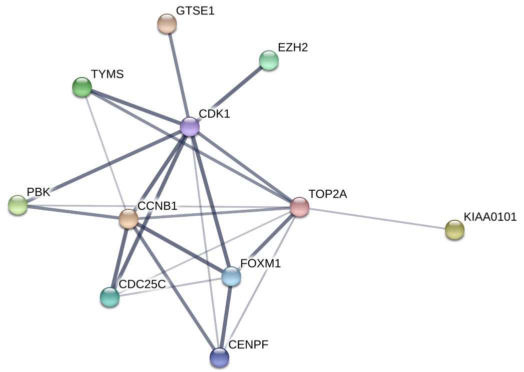

Cellular response to extracellular stimuli	
Nodes	12
Edges	23
Node degree	3.83
Clustering coefficient	0.862
PPI enrichment p-value	5.93e-11
