# Supplementary material for: Functional transcriptomic annotation and protein–protein interaction network analysis identify NEK2, BIRC5, and TOP2A as potential targets in obese patients with luminal A breast cancer
Source: Breast Cancer Res Treat. 2018 Jan 12;168(3):613–23. doi: 10.1007/s10549-017-4652-3 (PMC5842257; doi:10.1007/s10549-017-4652-3)
Supplement: Supplementary file 5 — Supplementary material 5 (PDF 52 kb) [file 10549_2017_4652_MOESM5_ESM.pdf]

Overexpressed genes (n=81)				*Underexpressed genes (n=96)*			
**ID**	**Gene**	**FC**	***p*-value**	**ID**	**Gene**	**FC**	***p*-value**
206204_at	GRB14	-2.479	0.001	201525_at	APOD	2.472	0.000
203999_at	SYT1	-2.228	0.000	206378_at	SCGB2A2	2.376	0.018
205358_at	GRIA2	-2.214	0.025	207147_at	DLX2	2.268	0.000
206325_at	SERPINA6	-2.074	0.006	209612_s_at	ADH1B	2.184	0.000
205440_s_at	NPY1R	-2.019	0.002	218730_s_at	OGN	2.117	0.000
205696_s_at	GFRA1	-1.934	0.000	207175_at	ADIPOQ	1.897	0.005
221577_x_at	GDF15	-1.801	0.000	206799_at	SCGB1D2	1.879	0.074
213712_at	ELOVL2	-1.687	0.005	202037_s_at	SFRP1	1.856	0.000
204286_s_at	PMAIP1	-1.683	0.000	209542_x_at	IGF1	1.782	0.000
213245_at	ADCY1	-1.640	0.007	219935_at	ADAMTS5	1.766	0.000
202708_s_at	HIST2H2BE	-1.631	0.002	217528_at	CLCA2	1.758	0.002
206457_s_at	DIO1	-1.631	0.051	202965_s_at	CAPN6	1.751	0.000
204825_at	MELK	-1.625	0.000	209242_at	PEG3	1.735	0.002
208305_at	PGR	-1.623	0.003	205529_s_at	RUNX1T1	1.732	0.000
218542_at	CEP55	-1.620	0.000	202992_at	C7	1.700	0.001
203413_at	NELL2	-1.620	0.020	214598_at	CLDN8	1.689	0.000
204595_s_at	STC1	-1.611	0.009	219768_at	VTCN1	1.687	0.050
219438_at	NKAIN1	-1.610	0.002	203373_at	SOCS2	1.686	0.000
210272_at	CYP2B7P	-1.605	0.022	212865_s_at	COL14A1	1.686	0.000
209773_s_at	RRM2	-1.591	0.000	204755_x_at	HLF	1.630	0.001
218755_at	KIF20A	-1.585	0.000	206143_at	SLC26A3	1.618	0.036
204623_at	TFF3	-1.577	0.011	209351_at	KRT14	1.615	0.028
202589_at	TYMS	-1.575	0.000	204363_at	F3	1.614	0.000
201292_at	TOP2A	-1.572	0.002	205830_at	CLGN	1.610	0.029
219412_at	RAB38	-1.550	0.003	205908_s_at	OMD	1.606	0.003
218009_s_at	PRC1	-1.547	0.000	214087_s_at	MYBPC1	1.605	0.064
204343_at	ABCA3	-1.527	0.000	209291_at	ID4	1.597	0.000
202705_at	CCNB2	-1.525	0.000	214321_at	NOV	1.592	0.000
218355_at	KIF4A	-1.522	0.000	214451_at	TFAP2B	1.589	0.171
219918_s_at	ASPM	-1.520	0.002	204712_at	WIF1	1.587	0.006
205380_at	PDZK1	-1.518	0.142	219140_s_at	RBP4	1.568	0.001
207267_s_at	RIPPLY3	-1.515	0.002	33767_at	NEFH	1.566	0.004
210559_s_at	CDK1	-1.506	0.001	213071_at	DPT	1.566	0.000
202095_s_at	BIRC5	-1.505	0.000	203290_at	HLA-DQA1	1.557	0.033
207828_s_at	CENPF	-1.505	0.000	207057_at	SLC16A7	1.543	0.001
221521_s_at	GINS2	-1.499	0.000	205828_at	MMP3	1.536	0.006
218510_x_at	FAM134B	-1.499	0.000	204748_at	PTGS2	1.536	0.004
204641_at	NEK2	-1.495	0.001	211737_x_at	PTN	1.536	0.003
210052_s_at	TPX2	-1.484	0.000	206201_s_at	MEOX2	1.533	0.001
207144_s_at	CITED1	-1.482	0.006	212762_s_at	TCF7L2	1.531	0.001
205167_s_at	CDC25C	-1.479	0.000	204948_s_at	FST	1.525	0.003
207165_at	HMMR	-1.479	0.001	205478_at	PPP1R1A	1.521	0.000
210523_at	BMPR1B	-1.478	0.025	203896_s_at	PLCB4	1.520	0.003
208016_s_at	AGTR1	-1.477	0.035	204749_at	NAP1L3	1.520	0.000
213599_at	OIP5	-1.476	0.000	204719_at	ABCA8	1.515	0.003
205862_at	GREB1	-1.472	0.004	211276_at	TCEAL2	1.510	0.003
207400_at	NPY5R	-1.471	0.008	209750_at	NR1D2	1.506	0.001
204379_s_at	FGFR3	-1.470	0.019	204679_at	KCNK1	1.506	0.032
204916_at	RAMP1	-1.468	0.003	205883_at	ZBTB16	1.497	0.005
205948_at	PTPRT	-1.467	0.028	204939_s_at	PLN	1.492	0.001
209408_at	KIF2C	-1.464	0.000	215311_at	NTRK3	1.490	0.006
202718_at	IGFBP2	-1.462	0.006	212451_at	SECISBP2L	1.486	0.002
202580_x_at	FOXM1	-1.461	0.000	206227_at	CILP	1.484	0.002
209642_at	BUB1	-1.461	0.000	206091_at	MATN3	1.476	0.010
222361_at	TUBBP5	-1.461	0.003	202747_s_at	ITM2A	1.475	0.002
203764_at	DLGAP5	-1.461	0.005	214807_at	PLXDC2	1.475	0.002
205483_s_at	ISG15	-1.460	0.007	215271_at	TNN	1.475	0.004
210367_s_at	PTGES	-1.460	0.002	209840_s_at	LRRN3	1.472	0.006
204545_at	PEX6	-1.459	0.001	202363_at	SPOCK1	1.472	0.001
221667_s_at	HSPB8	-1.458	0.057	213429_at	BICC1	1.471	0.001
218039_at	NUSAP1	-1.458	0.000	203548_s_at	LPL	1.469	0.003
212022_s_at	MKI67	-1.457	0.000	204154_at	CDO1	1.468	0.001
205590_at	RASGRP1	-1.457	0.016	212382_at	TCF4	1.468	0.004
202503_s_at	KIAA0101	-1.449	0.001	205239_at	AREG	1.464	0.071
214978_s_at	PPFIA4	-1.446	0.006	212188_at	KCTD12	1.463	0.000
204026_s_at	ZWINT	-1.445	0.000	212670_at	ELN	1.462	0.000
219148_at	PBK	-1.443	0.008	221583_s_at	KCNMA1	1.462	0.005
204475_at	MMP1	-1.443	0.081	202118_s_at	CPNE3	1.460	0.031
213906_at	MYBL1	-1.443	0.011	210002_at	GATA6	1.460	0.002
202954_at	UBE2C	-1.441	0.000	220122_at	MCTP1	1.459	0.003
209278_s_at	TFPI2	-1.439	0.123	210072_at	CCL19	1.457	0.031
203755_at	BUB1B	-1.438	0.000	219580_s_at	TMC5	1.457	0.042
203358_s_at	EZH2	-1.437	0.000	209763_at	CHRDL1	1.455	0.026
204318_s_at	GTSE1	-1.436	0.000	220005_at	P2RY13	1.453	0.000
219306_at	KIF15	-1.434	0.001	209335_at	DCN	1.450	0.001
214710_s_at	CCNB1	-1.431	0.000	201693_s_at	EGR1	1.450	0.007
209988_s_at	ASCL1	-1.431	0.187	220504_at	KERA	1.446	0.006
[illegible]	[illegible]	[illegible]	[illegible]	[illegible]	[illegible]	[illegible]	[illegible]
