# Supplementary material for: Functional transcriptomic annotation and protein–protein interaction network analysis identify NEK2, BIRC5, and TOP2A as potential targets in obese patients with luminal A breast cancer
Source: Breast Cancer Res Treat. 2018 Jan 12;168(3):613–23. doi: 10.1007/s10549-017-4652-3 (PMC5842257; doi:10.1007/s10549-017-4652-3)
Supplement: Supplementary file 8 — Supplementary material 8 (PDF 39 kb) [file 10549_2017_4652_MOESM8_ESM.pdf]

Function	K-M Plotter: Luminal A				K-M Plotter: Luminal B				K-M Plotter: HER2+				K-M Plotter: TNBC			
	RFS		OS		RFS		OS		RFS		OS		RFS		OS	
	HR	*p*-value	HR	*p*-value	HR	*p*-value	HR	*p*-value	HR	*p*-value	HR	*p*-value	HR	*p*-value	HR	*p*-value
**Cell cycle**	2.22(1.86-2.65)	0.000	2.71(1.85-3.97)	0.000	1.39(1.03-1.9)	0.033	1.27(0.64-2.49)	0.49	0.82(0.52-1.29)	0.38	1.22(0.56-2.68)	0.62	1.33(0.97-1.84)	0.08	0.5(0.25-0.97)	0.036
**Cell differentiation**	1.9(1.6-2.27)	0.000	2.36(1.62-3.34)	0.000	1.27(1.05-1.54)	0.015	1.31(0.9-1.9)	0.16	0.61(0.41-0.9)	0.011	0.93(0.48-1.77)	0.82	1.04(0.81-1.33)	0.78	0.82(0.5-1.34)	0.42
**Cell proliferation**	2.12(1.78-2.53)	0.000	2.73(1.86-4.01)	0.000	1.03(0.76-1.4)	0.85	0.99(0.5-1.93)	0.96	0.87(0.55-1.38)	0.56	0.99(0.45-2.18)	0.99	1.26(0.91-1.74)	0.16	0.49(0.25-0.97)	0.035
**Cellular response to EC stimuli**	1.93(1.62-2.3)	0.000	2.34(1.61-3.4)	0.000	1.24(0.91-1.69)	0.16	1.41(0.71-2.8)	0.32	0.65(0.41-1.03)	0.062	0.98(0.44-2.14)	0.95	1.18(0.86-1.63)	0.31	0.6(0.31-1.16)	0.13
