# Supplementary material for: Functional transcriptomic annotation and protein–protein interaction network analysis identify NEK2, BIRC5, and TOP2A as potential targets in obese patients with luminal A breast cancer
Source: Breast Cancer Res Treat. 2018 Jan 12;168(3):613–23. doi: 10.1007/s10549-017-4652-3 (PMC5842257; doi:10.1007/s10549-017-4652-3)
Supplement: Supplementary file 9 — Supplementary material 9 (PDF 22 kb) [file 10549_2017_4652_MOESM9_ESM.pdf]

Cell cycle		Cell differentiation		Cell proliferation		Cellular response to extracellular stimuli	
Node	*Edges*	*Node*	*Edges*	*Node*	*Edges*	*Node*	*Edges*
BUB1	27	CDK1	6	BUB1	15	CDK1	9
CDK	27	TOP2A	6	CCNB2	15	TOP2A	8
CCNB1	25	FOXM1	5	CDK1	15	CCNB1	7
CCNB2	25	CENPF	4	CCNB1	14	FOXM1	5
BIRC5	24	NEK2	4	BIRC5	12	CDC25C	4
KIF11	23			PRC1	12	CENPF	4
TOP2A	23			BUB1B	11		
PRC1	21			CENPF	10		
BUB1	19			DLGAP5	10		
DLGAP5	19			FOXM1	9		
KIF4A	19						
CENPF	18						
KIF20A	18						
NEK2	17						
CEP55	16						
MELK	16						
NUSAP	16						
UBE2C	15						
