# Supplementary material for: Functional transcriptomic annotation and protein–protein interaction network analysis identify NEK2, BIRC5, and TOP2A as potential targets in obese patients with luminal A breast cancer
Source: Breast Cancer Res Treat. 2018 Jan 12;168(3):613–23. doi: 10.1007/s10549-017-4652-3 (PMC5842257; doi:10.1007/s10549-017-4652-3)
Supplement: Supplementary file 10 — Supplementary material 10 (PDF 101 kb) [file 10549_2017_4652_MOESM10_ESM.pdf]

Gene	Source	DRUG	Mechanism of Action
**BIRC5**	DrugBank, ApexBio, DGIdb, and Novoseek	Docetaxel	Microtubulin disassembly inhibitor, Tubulin and VEGF inhibitor, Taxanes, Microtubule stabilizer
		Paclitaxel	Tubulin and Bcl2 inhibitor, Taxanes
		**LY2181308**	Survivin inhibitors
		**YM155**	Survivin suppressant, apoptosis inhibitor, Survivin inhibitors
		**YM-155 hydrochloride**	Potent survivin inhibitor
		AEG40826 **(**HGS10299)	IAP (Inhibitor of Apoptosis Protein) family inhibitor
		**SPC-3042**	Survivin inhibitor
**BUB1**	Novoseek and HMDB	Adenosine triphosphate	
		Adenosine diphosphate	Full agonist, Agonist
**CCNB1**	ApexBio and Novosek	Temozolamide	Alkylating Agents
		Nocodazole	Tubulin production inhibitor,anti-neoplastic agent
		Purvalanol A,	
		(R)-DRF053 dihydrochloride	cdk/CK1 inhibitor, potent and ATP-competitive
		Aminopurvalanol A	
		Bendamustine HCl	Cytostatic agent for non-Hodgkin lymphomas
		NSC 693868	
**CDK1**	DrugBank, PharmGKB, ApexBio, DGIdb, HMDB, Tocris, and Novoseek	Deferasirox	Oral iron chelator
		Alsterpaullone	Target, inhibitor
		Flavopiridol	Pan-cdk inhibitor
		Olomoucine	Target, binder
		SU9516	Target, binder
		Nocodazole	Tubulin production inhibitor,anti-neoplastic agent
		Hymenialdisine	Target
		Indirubin-3'-Monoxime	Target, binder
		AT7519	Multi-CDK inhibitor
		Aminopurvalanol A	
		**BMS265246**	CDK1/2 inhibitor,potent and selective, Potent cdk1/2 inhibitor
		Kenpaullone	Potent cyclin-dependent kinase inhibitor. Also inhibits GSK-3
		**Ro 3306**	An ATP-competitive, potent CDK1 inhibitor, Cyclin-dependent kinase (cdk) 1 inhibitor
		SCH727965	CDK inhibitor, CDK2 Inhibitors, Kinase Inhibitors
		radiotherapy	
		**I-DRF053 dihydrochloride**	cdk/CK1 inhibitor,potent and ATP-competitive
		AT7519 Hydrochloride	Multi-CDK inhibitor
		AT7519 trifluoroacetate	CDK/cyclin inhibitor
		**AZD-5438**	Potent CDK1/2/9 inhibitor
		AZD-5597	Potent CDK inhibitor
		BS-181 HCl	CDK7 inhibitor,highly selective
		CDK inhibitor **II**	
		CGP60474	CDKs and PKC inhibitor, potent
		Dinaciclib (SCH727965)	Potent CDK inhibitor

		Flavopiridol hydrochloride	
		LDC000067	
		NSC 693868	
		Nu 6027	ATR/CDK inhibitor, potent and selective
		**NVP-LCQ195**	CDK1/CDK2/CDK5 inhibitor
		**P276-00**	CDK-1/CDK4/CDK9 inhibitor
		PF 4800567 hydrochloride	Selective casein kinase 1eta inhibitor
		PHA-793887	Pan-Cdk inhibitor
		PHA-848125	CDK inhibitor, potent and ATP-competitive
		**Purvalanol B**	CDK1/CDK2/CDK4 inhibitor
		**R547**	CDK1/2/4 inhibitor,ATP-competitive
		TMCB	Dual-kinase inhibitor; inhibits CK2 and ERK8
		ALOISINE A	
		ALSTERPAULLONE 2-CYANOETHYL	
		alvocidib	Kinase Inhibitors
		AURORA KINASE/CDK INHIBITOR	
		BAY 1000394	pan-CDK inhibitor
		BOHEMINE	
		CDK1 INHIBITOR (CGP74514A)	
		CDK1/2 INHIBITOR III	
		CDK1/5 INHIBITOR	
		CDK2 INHIBITOR III	
		CDK2 INHIBITOR IV	
		CDK4 inhibitor	CDK4/Cyclin D1 inhibitor
		COMPOUND 52 [PMID:9677190]	
		GSK-3 INHIBITOR IX	
		RGB-286638	CDKs inhibitor
		RONICICLIB	
		Senexin A	CDK8 inhibitor, Cyclin-dependent kinase 8 (cdk8) inhibitor
**FOXM1**	ApexBio, Tocris, and Novoseek	Verteporfin	YAP inhibitor; disrupts YAP-TEAD interactions
		**Thiostrepton**	FOXM1 inhibitor
		BI 6015	HNF4α antagonist, Hepatocyte nuclear factor 4alpha (HNF4alpha) antagonist
		GANT61	GLI antagonist, GLI antagonist; inhibits Hedgehog (Hh) signaling
**KIF11**	DrugBank, ApexBio, DGIdb, Tocris, and Novoseek	Monastrol	Selective inhibitor of mitotic kinesin Eg5
		**GSK-923295**	CENP-E inhibitor,small-molecule, Kinesin protein inhibitors
		**Ispinesib(SB-715992)**	Kinesin spindle protein (KSP) inhibitor, High affinity and selective allosteric KSP inhibitor
		MPI-0479605	Mps1 inhibitor,selective and ATP competitive
		**SB743921**	Potent KSP inhibitor
		4SC-205	inhibitor
		ALN-VSP	
		**ARQ-621**	Kinesin protein inhibitors
		**ARRY-520**	Kinesin protein inhibitors
		**AZD4877**	Kinesin protein inhibitors

		**LY-2523355**	Kinesin protein inhibitors
		**MK0731**	Kinesin protein inhibitors
		**SB-743921**	Kinesin protein inhibitors
		Paprotrain	Reversible inhibitor of MKLP-2
**KIF20A**	ClinicalTrials and HMDB	chenodeoxycholic acid	Antagonist
		Guanosine triphosphate	
		Cathartics	
		Gastrointestinal Agents	
		Laxatives	
		Vaccines	
**KIF4A**	ApexBio	**Ispinesib (SB-715992)**	Kinesin spindle protein (KSP) inhibitor
		**SB743921**	Potent KSP inhibitor
**MELK**	ApexBio	Adenosine triphosphate	Full agonist, Agonist
		**OTSSP167**	MELK inhibitor
		**OTSSP167 hydrochloride**	MELK inhibitor, highly potent and selective
**PBK**	ApexBio, DGIdb, and HMDB	Adenosine triphosphate	
		OTS964	TOPK inhibitor, potent and selective
		OTS514	TOPK inhibitor, highly potent
**RRM2**	DrugBank, PharmGKB, ApexBio, DGIdb, HMDB, and Novoseek	Cladribine	Apoptosis inducer in CLL cells
		**Gemcitabine**	Ribonucleotide reductase and DNA synthesis inhibitor, Nucleoside Analogs
		Hydroxyurea	
		Gallium nitrate	
		**Clofarabine**	Antimetabolite, inhibit DNA polymerase and ribonucleotide reductase
		Cytarabine	
		Fludarabine	DNA synthesis inhibitor
		Adenosine triphosphate	
		CDP	
		Guanosine diphosphate	
		Trypanothione	
		Uridine-5’-Diphosphate	
		motexafin gadolinium	
		GTI 2040	
		Imexon	
		**Triapine**	Ribonucleotide reductase inhibitor, Others
		Fludarabine Phosphate (Fludara)	Inhibits STAT1 activation and DNA synthesis
		LOR-2040	
**TOP2A**	DrugBank, ClinicalTrials, ApexBio, DGIdb, FDA Approved Drugs, HMDB, Tocris, and Novoseek	**Etoposide**	Topo II inhibitor, Topoisomerase 2 Inhibitors, Topoisomerase II inhibitor
		**Doxorubicin**	Topo II inhibitor,3mmunosuppressive antineoplastic antibiotic
		**Teniposide**	inhibitor, Target
		**Daunorubicin**	DNA topoisomerase II inhibitor
		**Epirubicin**	inhibitor, Target
		**Amsacrine**	Topoisomerase 2 inhibitor
		Mitoxantrone	inhibitor, Target
		Ciprofloxacin	Target, inhibitor
		Ofloxacin	Fluoroquinolones, antibiotics
		Idarubicin	inhibitor, Target
		Podofilox	Target, inhibitor
		Valrubicin	inhibitor, Target
		**Doxil**	DNA intercalator and TOPO II inhibitor, Topoisomerase 2 Inhibitors

		Dexrazoxane	Target, inhibitor
		Enoxacin	Target, inhibitor
		Levofloxacin	Target, inhibitor
		Lomefloxacin	Target, inhibitor
		Lucanthone	Target, inhibitor
		Moxifloxacin	Target, inhibitor
		Norfloxacin	Target, inhibitor
		Pefloxacin	Target, inhibitor
		Sparfloxacin	Target, inhibitor
		Trovafloxacin	Target, inhibitor
		**Amrubicin**	Topoisomerase II inhibitor, anthracycline agent
		Cyclophosphamide	Nitrogen mustard alkylating agent and prodrug.
		Docetaxel	Microtubulin disassembly inhibitor, Tubulin and VEGF inhibitor, Taxanes, Microtubule stabilizer
		Paclitaxel	Tubulin and Bcl2 inhibitor, Taxanes
		Topotecan	Topoisomerase 1 inhibitor, TOPO I inhibitor, Topoisomerase 1 Inhibitors
		Trastuzumab	HER2 antagonist, HER2 Inhibitors, Therapeutic Antibodies, Epidermal growth factor receptor (EGFR) inhibitors
		Dactinomycin	Target
		Finafloxacin	Target, inhibitor
		Fleroxacin	Target, inhibitor
		Carboplatin	Antitumor agent that forms platinum-DNA adducts., Platinum
		Fluorouracil	RNA processing inhibitor and thymidylate synthase inhibitor
		Pertuzumab	HER2 Inhibitors, Therapeutic Antibodies, Epidermal growth factor receptor (EGFR) inhibitors, HER2 Dimerization Inhibitor
		Gatifloxacin	Fluoroquinolone antibiotic,inhibits bacterial TOPO II
		Irinotecan	Topoisomerase I inhibitor, TOPO I inhibitor, Topoisomerase 1 Inhibitors
		Avelox I.V.	
		Ellence	
		Valstar	
		Camptothecin	Topoisomerase I inhibitor,prototypic
		**Amonafide**	DNA intercalator,Topo II inhibitor
		Genistein	AR agonist
		RTA 744	Target
		Banoxantrone	Target
		Elsamitrucin	Target
		SP1049C	Target
		ZEN-012	Target
		**Pirarubicin**	Topo II inhibitor; antineoplastic; analogue of doxorubicin
		DTS-201	
		Flumequine	
		INNO-206	Prodrug of doxorubicin
		**Voreloxin**	Topo II inhibitor
		**ellipticine**	DNA topoisomerase II inhibitor
		Antimitotic Agents	

		(S)-10-Hydroxycamptothecin	
		**Amsacrine hydrochloride**	Topoisomerase 2 inhibitor
		Beta-Lapachone	DNA topoisomerase I inhibitor, selective
		**Dexrazoxane HCl (ICRF-187, ADR-529)**	Topoisomerase II inhibitor, intracellular ion chelator, cardioprotective agent
		**Doxorubicin (Adriamycin) HCl**	Antitumour antibiotic, inhibits TOPO II.
		Epirubicin HCl	Antibiotic antitumor agent
		Idarubicin HCl	Anthracycline and daunorubicin analog, topoisomerase inhibitor
		Mitoxantrone HCl	Topoisomerase II inhibitor, anti-neoplastic drug
		Moxifloxacin HCl	Fluoroquinolone antibiotic
		Novobiocin Sodium	
		Podophyllotoxin	
		Voreloxin Hydrochloride	Antineoplastic naphthyridine analogue
		AEZS-112, ZEN-012	
		BECATECARIN	
		C-1311	
		CAP7.1	
		CNDO101	
		GPX-100	
		Idronoxil	Caspase activator, XIAP inhibitor, FLIP suppressor, and TOPO II inhibitor
		topotecan hydrochloride	DNA topoisomerase I inhibitor; Camptothecin (Cat. No. 1100) analog
**NEK2**	DrugBank, ApexBio, HMDB, and Novoseek	Adenosine triphosphate	
		INH6	
		Magnesium	
**TYMS**	DrugBank, PharmGKB, ApexBio, DGIdb, FDA Approved Drugs, HMDB, Tocris, and Novoseek	**Pemetrexed**	TS, DHFR, GARFT and AICARFT inhibitor, DHFR and Thymidylate synthase inhibitor, Anti-Folates
		Raltitrexed	inhibitor, Target
		**Capecitabine**	RNA processing inhibitor and thymidylate synthase inhibitor, F-pyrimidines
		**Fluorouracil**	RNA processing inhibitor and thymidylate synthase inhibitor
		Methotrexate	Folate antagonist, inhibits DFHR
		Floxuridine	Antineoplastic antimetabolite, RNA and DNA synthesis inhibitor
		Tegafur	
		Gemcitabine	Ribonucleotide reductase and DNA synthesis inhibitor, Nucleoside Analogs
		leucovorin	
		Trifluridine	Target, inhibitor
		Trimethoprim	Target, inhibitor
		Irinotecan	Topoisomerase I inhibitor, TOPO I inhibitor, Topoisomerase 1 Inhibitors
		Pralatrexate	Antifolate, a folate analog, RFC-1 targeted DHFR inhibitor
		Alimta	
		Folotyn	
		Gemzar	
		Xeloda	
		Dihydrofolic acid	
		OSI-7904L	Target

		ANX-510	Target
		NB1011	Target
		Triacsin C	Inhibitor of acyl-CoA synthetase
		Pyrimidine analogues	
		Doxifluridine	
		Leucovorin Calcium	Derivative of folic acid
		N1,N12-Diethylspermine tetrahydrochloride	
		**Nolatrexed (AG-337**)	Non-classical thymidylate synthase inhibitor
		Pemetrexed disodium hemipenta hydrate	
		Risedronate Sodium	FPP synthase inhibitor
		Triflurdine (Viroptic)	Anti-herpesvirus antiviral drug
		MODUFOLIN	
		NB-1011	
		ONX 0801	
		AT 56	L-PGDS inhibitor
		Borrelidin	Antiangiogenic; inhibits threonyl-tRNA synthetase
		HQL 79	Human hematopoietic prostaglandin D synthase (H-PGDS) inhibitor
		PD 404182	High affinity inhibitor of KDO 8-P synthase; also DDAH1 inhibitor
**CDC25C**	ApexBio, HMDB, Tocris, and Novoseek	Temozolomide	Alkylating Agents
		Phosphoric acid	
		**NSC 95397**	Selective Cdc25 dual specificity phosphatase inhibitor
		NSC 663284	
**EZH2**	ApexBio, DGIdb, HMDB, and Novoseek	L-Lysine	Agonist
		S-Adenosylmethionine	
		s-adenosylhomocysteine	
		**EI1**	EZH2 inhibitor
		**EPZ-6438**	EZH2 inhibitor, potent and selective
		**GSK126**	EZH2 inhibitor
		**3-Deazaneplanocin A (DZNep) hydrochloride**	Cell permeable SAHH and ENZ2 inhibitor
		**CPI-169**	EZH2 inhibitor
		**EPZ005687**	EZH2 inhibitor, potent and selective
		**GSK343**	EZH2 inhibitor, potent, selective and cell permeable
		**GSK503**	EZH2 inhibitor
		**UNC1999**	EZH2 inhibitor
